# Trichostatin A-Induced H3K9ac Hyperacetylation Selectively Enriches Promoters in Adipogenic Human Periodontal Ligament Stem Cells

**DOI:** 10.3390/ijms27219898

**Published:** 2026-11-05

**Authors:** Julio A. Montero-Del-Toro, Angelica A. Serralta-Interian, Monica Lamas, David Valle-García, Julio C. Torres-Romero, Beatriz A. Rodas-Junco

**Affiliations:** 1 Facultad de Ingeniería Química, Universidad Autónoma de Yucatán, Mérida 97203, Mexico; a11003655@alumnos.uady.mx (J.A.M.-D.-T.); angelica.serralta@correo.uady.mx (A.A.S.-I.); 2 Laboratorio de Células Troncales, Facultad de Odontología, Universidad Autónoma de Yucatán, Mérida 97000, Mexico; 3 Departamento de Farmacobiología, Centro de Investigación y de Estudios Avanzados-Sede Sur, Ciudad de México 07360, Mexico; mlamas@cinvestav.mx; 4 Centro de Investigación sobre el Envejecimiento, Centro de Investigación y de Estudios Avanzados del I.P.N-Sede Sur, Ciudad de México 07360, Mexico; david.valle@cinvestav.mx; 5 Laboratorio de Bioquímica y Genética Molecular, Facultad de Química, Universidad Autónoma de Yucatán, Calle 43 s/n x 96 Paseo de las Fuentes y 40 Col. Inalámbrica, Mérida 97069, Mexico; julio.torres@correo.uady.mx; 6 SECIHTI-Facultad de Ingeniería Química, Universidad Autónoma de Yucatán, Mérida 97203, Mexico

**Keywords:** H3K9ac, chromatin remodeling, ChIP-seq, periodontal ligament stem cells, adipogenic differentiation, trichostatin A

## Abstract

Adipogenesis is regulated by histone modifications associated with chromatin accessibility. These modifications include histone H3 lysine 9 acetylation (H3K9ac), a mark linked to transcriptional activation. Its dynamics are governed by histone acetyltransferases and histone deacetylases (HDACs). Although trichostatin A (TSA) induces global hyperacetylation, its association with the differential enrichment of H3K9ac at regulatory elements during adipogenesis in human cells remains unclear. Therefore, this study characterized the genomic distribution of TSA-induced H3K9ac enrichment in human periodontal ligament stem cells (hPDLSCs). To this end, hPDLSCs underwent adipogenic differentiation for 14 days following TSA pretreatment (100 nM, 72 h). Cell morphology, lipid droplet radius, and the nuclear distribution of H3K9ac and H3K9me2 were evaluated by immunofluorescence, while the genomic distribution of H3K9ac was assessed by ChIP-seq. Statistical significance was assessed using an unpaired two-tailed Student’s *t*-test (*p* < 0.05) and MACS3 peak calling (q = 0.001). TSA reduced the lipid droplet radius by 1.69-fold and increased the nuclear H3K9ac signal by 10.17-fold, while reducing H3K9me2 signal by 1.38-fold. Furthermore, 752 promoters exhibited greater H3K9ac enrichment under TSA treatment compared with 364 in the control, including genes associated with adipogenesis, such as *PPARGC1B* and *AGPAT2*, and chromatin remodeling genes *SETD5* and *KDM2A*. Overall, these findings indicate that TSA alters the adipogenic phenotype and increases H3K9ac enrichment at promoters associated with metabolic and chromatin remodeling programs.

## 1. Introduction

Adipogenesis is a cellular differentiation process essential for adipose tissue homeostasis and plasticity [1]; its dysregulation is associated with obesity and type 2 diabetes. Adipogenesis requires coordinated changes in gene expression during adipogenic lineage commitment and specialization, involving epigenetic mechanisms such as histone post-translational modifications, particularly acetylation and deacetylation [2]. These dynamic modifications [3], along with structural alterations induced by DNA methylation at CpG islands [4], contribute to establishing chromatin states compatible with the activation or repression of transcriptional programs.

Adipogenic differentiation is a multifaceted cellular transformation accompanied by distinct morphological and phenotypic features, including mitotic clonal expansion of preadipocytes, cell cycle reorganization, cytoskeletal remodeling, and intracellular lipid droplet accumulation [2]. At the epigenetic level, this transition is governed by extensive chromatin alterations across multiple histone residues, involving diverse acetylation marks on the H3 and H4 tails, such as H3K14ac, H3K18ac, H3K56ac, and H4K16ac, as well as crosstalk with lysine methylation [5,6]. Within this landscape, histone H3 lysine 9 acetylation (H3K9ac) functions as a critical epigenetic switch, with H3K9ac and H3K9me2/me3 exerting directly opposing roles, and the balance between these marks influences the transcriptional state of genes involved in this process [2]. H3K9ac directly shifts this state toward open, permissive chromatin and transcriptional activation [7]. Quantitative mass spectrometry analyses demonstrate occupancy at this residue, whereby increased H3K9ac coincides with a marked decrease in the repressive H3K9me3 mark [5,6], supporting H3K9ac as a primary functional determinant of chromatin opening rather than a passive correlate. The dynamic distribution of H3K9ac is regulated by the balance between the activities of histone acetyltransferases (HATs) and histone deacetylases (HDACs) and is associated with transcriptional activation. Integrative approaches such as chromatin immunoprecipitation followed by high-throughput sequencing (ChIP-seq) enable the identification of genomic regions associated with promoters and candidate genes involved in transcriptional activation [7,8]. Although the role of H3K9ac in adipogenesis has been documented, much of the available evidence derives from functional studies in murine models, which have revealed dynamic reorganization of regulatory regions and histone modifications during differentiation [7,8]. However, the genomic organization of H3K9ac remains poorly characterized in human adult stem cells [9].

Human periodontal ligament stem cells (hPDLSCs) provide a suitable model for studying adipogenesis from an epigenetic perspective due to their higher adipogenic differentiation capacity [10,11]. Epigenetic mechanisms in adipocytes [12] and dental stem cells [13], along with the effects of pharmacological HDAC inhibition [14,15], have been investigated. Specifically, prior work by Serralta-Interian et al. [15] demonstrated that TSA (100 nM, 72 h) pretreatment enhances adipogenic differentiation and is accompanied by the transcriptional upregulation of canonical adipogenic markers such as *PPARγ* and *ADIPOQ*. Montero-Del-Toro et al. [16] subsequently established the temporal profile of the H3K9ac protein under the same TSA treatment conditions, revealing a prominent peak at day 14 that coincides with the transcriptional expression of key adipogenic regulators such as *PPARγ-2* and *C/EBPβ*. Based on this kinetic profile, day 14 was selected for genome-wide mapping because it captures an active chromatin state associated with adipogenic commitment, while avoiding both the acute, nonspecific pharmacological accumulation observed at day 0 and the stabilized epigenome of mature cells at day 28 [16], ultimately culminating in enhanced lipid deposition. Although classical adipogenic protocols typically assess phenotypic endpoints at 21–28 days, profiling the epigenome at these later stages would primarily capture a stabilized chromatin state rather than active repatterning of H3K9ac. Therefore, day 14 represents a critical transitional window for characterizing epigenetic priming during lineage commitment, prior to advanced phenotypic maturation.

Furthermore, TSA modifies global histone acetylation and methylation profiles [5] and has been identified as a direct promoter of adipogenic differentiation through specific H3K9ac changes [17]. However, an overall nuclear increase in H3K9ac does not necessarily indicate selective enrichment of this modification at specific genomic regions. To address this, TSA treatment was used as an H3K9ac hyperacetylation model, characterized by an increase in this modification through HDAC inhibition. This model allowed us to evaluate whether widespread H3K9ac elevation was associated with changes in its genome-wide distribution, particularly at promoter regions and other regulatory elements associated with adipogenic genes in hPDLSCs. For this purpose, ChIP-seq can characterize the genomic distribution of histone modifications and compare their enrichment between conditions [18], facilitate the association of regulatory regions with candidate target genes [8], identify DNA sequence patterns associated with transcription [19], and characterize regulatory features such as CpG islands [20].

Therefore, we hypothesized that TSA pretreatment modulates H3K9ac enrichment across specific genomic regions associated with adipogenesis. To test this hypothesis, we asked whether TSA-induced hyperacetylation leads to a selective enrichment of H3K9ac at promoters associated with adipogenic genes or to a uniform genomic increase without regional specificity. Phenotypic changes and the nuclear distribution of H3K9ac and H3K9me2 were examined by immunofluorescence, and the genomic enrichment of H3K9ac was assessed by ChIP-seq. Analysis of differentially enriched regions, their genomic distribution, and proximity-based gene associations enabled the identification of regulatory elements associated with the response to TSA and the prioritization of candidate regions and genes for future studies.

## 2. Results

### 2.1. Effect of TSA on hPDLSC Adipogenesis

To evaluate the effect of TSA-induced hyperacetylation on adipogenesis, cell morphology was analyzed after 14 days of adipogenic induction in control and TSA-pretreated (100 nM, 72 h) cells. Both conditions exhibited intracellular lipid droplet formation, characteristic of adipogenic differentiation (Figure 1A). The adipogenic identity of these structures was supported by the induction protocol previously validated by our group, which specifically promotes the accumulation of neutral lipid droplets [11,15,16]. At this early stage of differentiation, both control and TSA-pretreated cells displayed comparable overall lipid loads; however, they differed markedly in droplet size and distribution (Figure 1B,C). In control cells, lipid droplets were sparsely distributed, with a mean radius of 3.44 ± 1.98 μm (Figure 1B). In contrast, the TSA-pretreated cells exhibited smaller, clustered droplets, with a mean radius of 2.04 ± 0.49 μm. The number of droplets per cell was higher in TSA-pretreated cells (range, 41–241) than in control cells (range, 35–126) (Figure 1C). To determine whether these morphological differences were associated with changes in H3K9ac, immunofluorescence was used to evaluate its nuclear distribution.

### 2.2. TSA Alters the Nuclear Distribution of H3K9ac and H3K9me2 During hPDLSC Adipogenesis

We first assessed the nuclear distribution of H3K9ac after 72 h of hPDLSC exposure to examine inhibitor effectiveness. Immunofluorescence revealed a nuclear H3K9ac signal under both conditions. Vehicle-treated cells displayed discrete, low-intensity foci distributed throughout the nucleoplasm (Figure 2A, a–c), averaging 7.42 arbitrary fluorescence units (AFU). In contrast, TSA exposure was associated with a more intense and homogeneous H3K9ac signal, increasing the average to 9.55 AFU relative to the control (Figure 2A, d–f).

We then evaluated acetylation retention at the end of induction (day 14). The activating mark H3K9ac and the antagonistic mark H3K9me2 were analyzed in parallel. In control cells, the H3K9ac signal was confined to multiple focal domains throughout the nucleus (Figure 2B, a–c), averaging 1.40 AFU. In contrast, in TSA-treated cells, the fluorescent signal was more intense and homogeneously distributed (Figure 2B, d–f), reaching a mean of 14.24 AFU (Figure 2D).

For H3K9me2, an inverse pattern was observed. In control cells, discrete fluorescent foci were detected (Figure 2B, g–i) with a value of 0.44 AFU per nucleus. These foci were attenuated following inhibitor exposure, dropping to an average of 0.32 AFU (Figure 2E).

TSA exposure was associated with sustained epigenetic remodeling in hPDLSCs, with a global enrichment of H3K9ac. The distribution of this mark was analyzed by ChIP-seq to determine its genomic impact.

### 2.3. Quality Metrics and Alignment Validation

Initial quality evaluation and alignment metrics were used to assess the ChIP-seq libraries. Following read deduplication, the libraries retained 67.91% unique reads, indicating the observed library complexity. Mapping to the human reference genome (hg38) yielded 37,003,242 mapped reads (64.25% mapping rate) for the basal condition and 26,008,942 mapped reads (54.32% mapping rate) for the TSA condition. To improve comparability of sequencing depth prior to differential enrichment analysis, the basal condition was downsampled to approximate the mapped read depth of the TSA library, resulting in 25,906,266 mapped reads (64.26% mapping rate). Further quality assessment confirmed robust genome-wide enrichment, identifying 31,593 peaks for the original basal condition (24,749 peaks after downsampling) and 27,283 peaks for the TSA condition, with fraction of reads in peaks (FRiP) scores of 19.62% and 23.90%, respectively.

### 2.4. Genomic Distribution and Gene Association of H3K9ac-Enriched Regions

We evaluated the genomic distribution of H3K9ac-enriched regions. In both conditions, enriched regions were located primarily at proximal promoters (≤1 kb from the transcription start site [TSS]), followed by introns, exons, and distal intergenic regions (Figure 3A). Promoters accounted for 49.74% of the annotated peaks in the control condition and 55.10% in the TSA condition, whereas distal intergenic regions represented 8.93% and 9.75%, respectively (Figure 3A). The H3K9ac signal was concentrated around the TSS in both conditions, with a higher enrichment in the TSA condition, whereas the input exhibited background signal levels across the assessed interval (Figure 3B). A chi-square test indicated that the distribution was nonrandom (*p* < 0.000001; Table S1), with enrichment observed at regulatory regions. To identify genes proximally associated with H3K9ac-enriched regions, peaks from each condition were analyzed using the Genomic Regions Enrichment of Annotations Tool (GREAT). Most enriched regions were associated with at least one gene in both the vehicle and TSA conditions, corresponding to 92.7% and 94.4% of the regions, respectively. Consequently, only 7.3% and 5.6% of the regions in the vehicle and TSA conditions, respectively, lacked gene associations under the model’s criteria (Figure 4A,D).

In both conditions, the highest proportion of associations occurred between 50 and 500 kb from the TSS, representing 36.0% of associations in the vehicle condition and 46.0% in the TSA condition. This was followed by associations located between 5 and 50 kb from the TSS (34.7% and 37.2% in the vehicle and TSA conditions, respectively), whereas associations >500 kb were less frequent (Figure 4B,E). The relative distance to the TSS displayed a comparable pattern between conditions, with most region–gene associations located between 5 and 500 kb downstream of the TSS (Figure 4C,F). Because this analysis encompassed all H3K9ac-enriched regions, we next evaluated the subset of differentially enriched promoters between conditions.

### 2.5. Differentially Enriched H3K9ac Promoters and Functional Characteristics of Associated Genes

We analyzed the subset of peaks located at promoter regions to identify differences in H3K9ac enrichment using differential enrichment analysis. Unlike the preceding CPM-normalized analyses, this approach requires comparable mapped read counts. Thus, the control condition was downsampled to 25,906,266 reads to approximate the 26,008,942 reads in the TSA condition, as described in the Methods. This approach was used to identify regions in which the H3K9ac signal differed between conditions. When located at promoters, these regions represent physical markers of changes in transcriptional potential.

Comparison of the TSA and control conditions identified a total of 13,644 H3K9ac-enriched promoters across the genome. Of these, 12,528 were enriched in both conditions, whereas 364 and 752 exhibited differential enrichment in the control and TSA conditions, respectively (Figure 5A; Table S3). Thus, 752 promoters showed higher H3K9ac enrichment in the TSA condition. A heatmap (±2.0 kb window) showed the highest signal near the center of differentially enriched promoter regions in both conditions. More differentially enriched promoter regions were identified in the TSA condition than in the control condition (Figure 5B).

Of the 752 promoters differentially enriched in the TSA condition, 311 were associated with protein-coding genes. To explore the biological functions of this gene set, functional enrichment analysis was performed using ShinyGO (Table S1). To reduce statistical inflation associated with the use of whole-genome reference datasets, a custom background comprising 12,304 genes, corresponding to all H3K9ac-enriched promoters detected in hPDLSCs (13,644 sequences), was used. Using this context-specific background, the analysis identified significant enrichment of several metabolic processes associated with adipogenic differentiation. The most strongly enriched biological categories included oxidative phosphorylation, cellular respiration, proton transmembrane transport, and ATP synthesis. This transition toward a high-mitochondrial metabolic phenotype is consistent with the energetic requirements of adipocyte maturation and lipid deposition (Figure 5C).

Because these processes included genes linked to differentially enriched promoters, their functional interactions were assessed using a protein–protein interaction network in STRING. The network comprised 311 proteins and exhibited a modular topology. Dense, interconnected clusters were identified (Figure 5D), including highly connected proteins such as MAPK1, which acts as an integrating node. The network included proteins associated with metabolic and signaling processes relevant to adipogenesis in hPDLSCs. Submodules associated with lineage commitment (WNT10B, FZD3, and BMPR2), mitochondrial biogenesis, and lipid metabolism (PPARGC1B) were identified. Furthermore, the glucose transporter SLC2A4 (GLUT4), associated with the mature adipogenic phenotype, was present in the network. The network also revealed a densely connected submodule of mitochondrial components, including *MT-ATP6*, *MT-ATP8*, *MT-CO1*, *MT-CO2*, *MT-CO3*, *MT-CYB*, *MT-ND1*, *MT-ND2*, *MT-ND3*, *MT-ND4*, *MT ND4L*, *MT-ND5*, and *MT-ND6*. Although these structural mitochondrial elements reflect bioenergetic reorganization and increased metabolic demand, the analytical relevance of the network rests on the nuclear regulators associated with this accelerated metabolic rate. Below, the most important genes linked to this process will be described in detail, briefly addressing their local H3K9ac distribution.

### 2.6. Local Configurations of H3K9ac Enrichment at Genes Related to Adipogenesis, Energy Metabolism, and Chromatin Remodeling

To examine the local H3K9ac distribution, a representative panel of 14 loci with differential enrichment under TSA treatment was selected, including 10 loci prioritized based on their functional relevance in the STRING network (*CTTN*, *TLN1*, *PPARGC1B*, *SETD5*, *SLC2A4*, *MEAF6*, *MBD3*, *EBF2*, *WNT10B*, and *AGPAT2*), as well as four additional loci selected based on their statistical weight in the differential analysis (*AAMDC*, *VSTM2A*, *ELOVL7*, and *LMF1*). The selection encompassed three interdependent functional categories: chromatin remodeling, adipogenesis, and energy metabolism. To confirm that the differential classification of these loci reflects a true quantitative change rather than an artifact of the analytical detection threshold, we extracted Log2 fold-change (LFC) values from normalized quantitative tracks at their promoter regions. This quantitative evaluation showed a continuum of positive H3K9ac enrichment (Table S2).

Inspection of binding profiles across these 14 loci revealed heterogeneity in local mark distribution, even among genes within the same functional category. Based on signal topology, three descriptive enrichment architectures were recognized (Figure 6): (i) dominant focal peak architecture, (ii) segmented enrichment architecture, and (iii) broad enrichment domains.

The first group of loci exhibited a dominant focal peak architecture, defined here as discrete enrichment regions ≤ 1.5 kb in width [21,22], in which the predominant enrichment signal remained sharply restricted to the 5′ end of the gene despite TSA-associated hyperacetylation. This pattern was observed in *AAMDC*, *SETD5*, *ELOVL7*, *MEAF6*, *EBF2*, and *TLN1*, with the main signal located at the promoter or early intragenic regions (Figure 6A). In *AAMDC*, which exhibited an LFC of 0.89 at its promoter, the strongest enrichment was concentrated over the promoter and first intron, coinciding with a dense CpG island, a promoter-like signature (PLS) element annotated by the Encyclopedia of DNA Elements (ENCODE), and enhancer-like elements in the same region. In contrast, the remainder of the gene body showed only weak secondary signals. *SETD5* and *ELOVL7*, with promoter LFC values of 0.79 and 1.11, respectively, displayed profiles dominated by a main promoter peak, accompanied by lower-intensity secondary signals across each locus. In *MEAF6*, which had a promoter LFC of 0.87, three main peaks clustered near the promoter were flanked by lower-intensity secondary signals. 5′-dominant profiles were also identified in *EBF2* and *TLN1*, which displayed promoter LFC values of 0.52 and 1.07, respectively. *TLN1* also exhibited a second enriched region toward the 3′ end of the gene, accompanied by an additional CpG island and separated from the promoter enrichment by an extensive intragenic region with lower-intensity signals. This focal pattern suggests a predominance of proximal transcriptional initiation mechanisms.

A second group of loci displayed a segmented enrichment architecture, operationally defined as supragenic structures > 10 kb in which multiple discrete, well-defined peaks are interconnected across permitted gaps along the locus [23]. This pattern was observed in *PPARGC1B*, *WNT10B*, *SLC2A4*, and *VSTM2A* (Figure 6B). In these genes, TSA-induced enrichment was not exclusively limited to the 5′ end but was organized into consecutive enriched regions separated by lower-signal zones. Although the signal was modular, the primary promoter regions retained a clear quantitative gain: *PPARGC1B* and *SLC2A4* had promoter LFC values of 0.81 and 1.60, respectively, accompanied by discrete intragenic signals along the gene body. *WNT10B* exhibited a promoter LFC of 1.55 while displaying additional prominent peaks distributed across the locus, spanning regulatory and coding elements. For *VSTM2A*, the signal was distributed across a series of sharp, independent enriched regions extending from the core promoter into adjacent regions. The promoter exhibited the highest LFC in this panel at 1.83. This distribution is consistent with a potential regulatory landscape that may be mediated by additional regulatory elements acting in coordination with the main promoter.

Finally, the third group exhibited broad enrichment domains, classified as uninterrupted signal regions spanning >1.5 kb to 10 kb, which denote sustained transcriptional elongation and serve as markers of lineage identity [21,22]. In these domains, the H3K9ac signal was distributed continuously, spanning large portions of the locus through a series of consecutive enriched regions of comparable intensity that did not return to baseline. The definitions used are based on peak width parameters, which are mark-agnostic and applicable to any histone modification, although the original references were based on H3K4me3 breadth.

This architecture was evident in the *LMF1*, *CTTN*, *MBD3*, and *AGPAT2* loci (Figure 6C). In these genes, increased acetylation formed extensive blocks covering promoter regions, CpG islands, and broad intragenic zones. Even within these continuous domains, quantitative extraction showed positive LFC values for the primary promoters, yielding values of 0.47, 1.04, 1.13, and 1.27 for *LMF1*, *CTTN*, *MBD3*, and *AGPAT2*, respectively. Unlike focal configurations, this architecture was characterized by a broader distribution across the gene body, which may be associated with an extended permissive chromatin state in genes with metabolic or structural functions demanding sustained transcriptional regulation during adipogenic differentiation.

## 3. Discussion

The acquisition of a new cellular identity requires extensive epigenetic reorganization to establish a specific differentiation program. Our findings show that TSA pretreatment was associated with changes in both the nuclear levels and genome-wide distribution of H3K9ac. Specifically, TSA was associated with an early increase in nuclear H3K9ac signal in hPDLSCs that persisted through day 14 of adipogenic differentiation, accompanied by a reduction in H3K9me2 signal and alterations in lipid droplet organization. At the genomic level, the selective enrichment of 752 promoters under TSA, compared with 364 in the control, indicates that TSA-induced hyperacetylation is not merely a global phenomenon but a targeted epigenetic reorganization. These results support our working hypothesis that TSA pretreatment is associated with differential H3K9ac enrichment at specific regulatory regions associated with adipogenic commitment, rather than solely reflecting a uniform genomic increase. This pattern aligns with previous studies in the same cellular model, in which exposure to HDAC inhibitors enhanced the adipogenic response and was accompanied by sustained increases in H3K9ac [14,15,16]. Additionally, TSA has been associated with greater lipid deposition by day 28 of induction in this cellular model [16]. Rather than assessing terminal maturation endpoints, genomic mapping at day 14 was intended to capture an active chromatin state associated with adipogenic commitment, while avoiding the acute, nonspecific pharmacological accumulation previously documented immediately following exposure [16].

Although this exploratory ChIP-seq framework identified chromatin-level associations rather than providing direct evidence of transcriptional proof warranting future orthogonal expression validations, this dynamic supports the notion that these modifications do not represent a static pharmacological response but instead depend on the biological context and stage of differentiation, as previously reported during the neuronal differentiation of human embryonic stem cells (HUES-9) [24].

At the genomic level, TSA-induced hyperacetylation coincided with region-specific differences in H3K9ac enrichment while maintaining the predominance of enrichment at promoter regions proximal to the TSS. This pattern is consistent with H3K9ac as a mark associated with transcriptionally active promoters [7]. These findings are also consistent with observations during the early stages of adipogenesis in murine 3T3-L1 preadipocytes, where H3K9ac has been implicated in establishing the differentiation program and in recruiting regulatory factors (p300/MED1) associated with lineage memory [25]. Beyond proximal regions, the identification of differentially enriched sequences within intergenic and intronic regions in the genomic profile of hPDLSCs suggests potential changes in the relationships between promoters and regulatory elements [26]. In other cellular systems, similar distal regions participate in chromatin interactions mediated by complexes such as Mediator and cohesin, whose co-occupancy is associated with chromatin loops connecting enhancers with lineage-specific promoters [27]. Therefore, the region-specific H3K9ac enrichment observed in this study may mark regulatory elements potentially involved in transcriptional control during adipogenesis.

Locus-level analysis revealed that epigenetic reorganization occurred across markedly heterogeneous local architectures, even among genes involved in analogous biological processes. The distribution of these focal, segmented, and broad domains may reflect the diversity of cCREs that differentially modulate the local accumulation of the mark [28]. These architectural variations further highlight the dependence of chromatin organization on the specific structural context of each locus, including features such as CpG island density [20]. Broad domains have been associated with housekeeping genes requiring constitutive expression. In contrast, focal or segmented patterns have been reported in genes involved in dynamic or stimulus-dependent regulation in response to adipogenic signals, hyperglycemia, or mechanical stress [29,30]. This topological diversity is consistent with the requirement for distinct levels of epigenetic permissiveness during the activation of the adipogenic transcriptional network thereby adding complexity to the individual regulation of each gene.

Consequently, this early organization of the epigenomic landscape was reflected in measurable structural changes in lipid droplets at day 14 of differentiation. Lipid droplet architecture is dynamic and can reflect the metabolic and functional state of the cell, with morphometry changing in response to processes such as lipogenesis and lipolysis [31,32,33]. The distinct morphometric profile observed at day 14 under TSA treatment, characterized by a higher number of smaller lipid droplets, warrants specific consideration. The total lipid content was not quantified in this study. TSA-treated cells displayed a distinct multilocular organization. In standard 2D in vitro models, the mechanical constraints imposed by flat substrates can restrict the formation of classical unilocular adipocytes [34,35]. Moreover, contemporary biophysical models of lipid droplet dynamics indicate that a multilocular phenotype is not necessarily indicative of developmental arrest. Rather, reduced droplet radius (from 3.44 ± 1.98 µm in controls to 2.04 ± 0.49 µm under TSA) and increased droplet numbers provided a greater interfacial surface area, a feature that has been associated with rapid *de novo* lipid synthesis and high metabolic demand [36,37]. Thus, rather than necessarily reflecting an inability to undergo fusion, the multilocular organization observed here may be consistent with an accelerated lipogenic state. Importantly, this transient structural organization aligns with the epigenetic enrichment profile captured by our ChIP-seq data, where hyperacetylation is enriched at lipid biogenesis networks, highlighting processes of oxidative phosphorylation, cellular respiration, ATP synthesis, and a component comprising multiple mitochondrial components and electron transport chain-related transcripts (such as *MT-ATP6*, *MT-CO1*, and members of the *MT-ND* gene family). The prominent representation of these components reflects the profound bioenergetic reorganization and mitochondrial biogenesis required to sustain the accelerated metabolic rate and functional demands of early adipogenic maturation in hPDLSCs.

These TSA-induced morphometric differences suggest a potential biological relationship to the differential H3K9ac enrichment identified at loci encoding transcriptional regulators and metabolic enzymes. Among these, *VSTM2A* is of particular interest because of its reported role during the early stages of adipogenic commitment. *VSTM2A* encodes a protein that is secreted early by preadipocytes and modulates cellular signaling, whereas its deficiency promotes adipocyte hypertrophy and disrupts lipid homeostasis [38,39]. Similarly, *PPARGC1B* encodes a transcriptional coactivator that coordinates metabolic programs, including mitochondrial biogenesis, oxidative phosphorylation, thermogenesis, and lipolytic processes [40]. Its activity has been implicated in differentiation in mesenchymal stem cells, where it functions as a transcriptional regulator of triglyceride accumulation and the expression of mature adipogenic markers, such as *PPARγ* and *FABP4*, thus contributing to the consolidation of cellular fate [41,42]. Changes in *PPARGC1B* expression have been associated with alterations in mitochondrial function and thermogenesis [43], which suggests that its early H3K9ac enrichment at this locus may be related to the biogenic dynamics favoring the formation of smaller lipid droplets observed after TSA treatment.

Similarly, increased H3K9ac enrichment at transcriptional regulatory elements [44] may be associated with the structural differences observed in lipid droplet distribution following TSA treatment at this developmental stage. These potential links between epigenetic regulation and the cellular phenotype can also be considered in the context of genes associated with lipid biogenesis. The *AGPAT2* gene encodes an enzyme essential for triacylglycerol biosynthesis; its loss of function inhibits lipid storage and compromises normal adipocyte formation [45], with alterations in its regulation reported in cancer cells [46]. Local H3K9ac enrichment at *AGPAT2* identifies this locus as a putative regulatory involvement that may contribute to explaining the greater number and smaller equivalent radius of lipid droplets induced by TSA. Likewise, *ELOVL7* participates in lipid remodeling through the metabolism of long-chain saturated fatty acids involved in the synthesis of neutral lipids such as cholesteryl esters [47]. *AAMDC* participates in early adipogenic maturation by promoting insulin-independent lipid storage [48]. Its expression is regulated post-transcriptionally by microRNAs [49] and, in tumor models, through signaling pathways such as PI3K–AKT–mTOR [50].

Taken together, differential H3K9ac enrichment at these loci suggests an epigenetically permissive state for lineage-identity pathways at this critical peak of the epigenetic profile, thereby modulating the early structural organization of adipocytes before terminal maturation. An important consideration when interpreting these results is that TSA does not act specifically on H3K9ac. As a class I and IIa HDAC inhibitor, TSA can simultaneously alter the acetylation of other residues within the H3 and H4 tails, such as H3K14, as well as aspects of chromatin organization, including nucleosome positioning and DNA accessibility [51]. In addition, classical HDACs have non-histone substrates, including p53, NF-kB, SP1, and members of the KLF family; thus, their effects are not restricted to chromatin structure [52,53]. In hPDLSCs, pretreatment with 100 nM TSA has also been associated with changes in the expression of *HDAC1*, *HDAC2*, and *HDAC3*, accompanied by alterations in *p300* and *GCN5* during differentiation [15].

In this context, changes in H3K9ac enrichment identified by ChIP-seq should be interpreted within the broader epigenetic response to TSA treatment. The present analysis characterizes differences in H3K9ac occupancy patterns under HDAC inhibition but does not establish the contribution of other histone modifications, nucleosome organization, or regulatory proteins. Therefore, the resulting profiles represent an H3K9ac-specific readout of the chromatin state associated with TSA treatment, rather than an integrated measure of all molecular effects of the treatment. This targeted mapping nonetheless provides an H3K9ac-specific framework for investigating potential relationships between the day 14 checkpoint and terminal lipid deposition. Although canonical adipogenic markers (such as *PPARγ*, *ADIPOQ*, *PPARγ-2*, and *C/EBPβ*) under identical TSA conditions were previously validated by our group [15,16], the primary objective of this work was to characterize the genomic distribution of H3K9ac. Consequently, rather than providing direct functional evidence, the targeted epigenetic mapping presented here provides an epigenetic roadmap linking this day 14 checkpoint to the terminal lipid deposition previously reported at day 28 [18]. This study has several limitations that should be explicitly acknowledged when interpreting the findings. Because this study focused on assessing the epigenetic activation state, the results should be interpreted in light of certain technical limitations. First, genome-wide ChIP-seq analyses were performed using a single biological sample per experimental group, precluding a direct assessment of biological variability. In addition, spike-in normalization was not performed; therefore, CPM normalization and downsampling cannot quantify absolute global changes in H3K9ac, and the coverage profiles should be interpreted as relative enrichment landscapes rather than as absolute measurements. Although biological replication with IDR analysis represents the standard for large consortium mapping, consortium criteria are not intended to be applied rigidly to all studies. To maximize analytical rigor under these constraints, we applied stringent peak-calling parameters, achieved high sequencing depth, assessed library complexity (NRF ≥ 0.8), and used matched input controls. Second, the hPDLSCs were derived from a single donor and cultured under standard 2D conditions, which may limit the generalizability of the findings. Therefore, the ChIP-seq results should be interpreted as initial, hypothesisgenerating genomic mapping rather than as confirmatory evidence of differential enrichment. Future studies with independent biological replicates and orthogonal validation (e.g., RNA-seq and ChIP-qPCR) will be required to confirm the candidate regions identified here and assess their functional relevance. Additionally, TSA is a nonspecific inhibitor with activity against several class I and IIa histone deacetylases. Thus, the global effects observed cannot be attributed exclusively to H3K9ac. Although orthogonal validation techniques were not integrated into the present study, this initial approach provides an exploratory basis for future experimental investigations. Collectively, these limitations define the scope within which the present conclusions should be interpreted, while supporting the value of this dataset as an initial epigenetic framework for future functional studies in hPDLSC adipogenesis.

## 4. Materials and Methods

### 4.1. Cell Culture

This study was conducted with informed consent from one donor and was approved by the Institutional Ethics Committee for Research of the Universidad Autónoma de Yucatán (No. CIE-06-2017). The hPDLSCs were derived from a stem cell line previously characterized by Montero-Del-Toro et al. [16]. Cells were thawed and initially seeded in T25 culture flasks containing α-MEM (Gibco, Grand Island, NY, USA) supplemented with 10% FBS (Gibco, Grand Island, NY, USA) and 1% penicillin-streptomycin (Gibco, Grand Island, NY, USA) and maintained at 37 °C and 5% CO_2_ in a humidified atmosphere. For subsequent expansion, cells were passaged into T75 flasks at a density of 7 × 10^4^ cells and used at passages 6–8.

### 4.2. Adipogenic Differentiation Under TSA Treatment

hPDLSCs were pretreated with 100 nM TSA (Sigma-Aldrich, St. Louis, MO, USA) for 72 h or with the vehicle (control group). A 1 mg/mL TSA stock solution prepared in sterile DMSO was diluted in culture medium to a final TSA concentration of 100 nM, with a final DMSO concentration of ≤0.1% (*v*/*v*). Vehicle controls received an equal volume of DMSO. These specific conditions were established based on our previous standardization studies in this cellular model, which showed that these conditions maintained cell viability and enhanced adipogenic differentiation [15,18]. Subsequently, the medium was replaced with adipogenic induction medium comprising α-MEM supplemented with 10% FBS, 1% antibiotic-antimycotic, 1.7 μM insulin, 1 μM dexamethasone, 500 μM 3-isobutyl-1-methylxanthine, and 60 μM indomethacin (Sigma-Aldrich, St. Louis, MO, USA), and the cells were maintained for 14 days. The 14-day induction period was selected for genomic mapping because previous findings indicated that this time point corresponds to the transient peak of global H3K9ac hyperacetylation [19]. The neutral lipid nature of the intracellular droplets accumulated under these standardized conditions was previously validated by Oil Red O histochemical staining using identical isolation and differentiation protocols [11,15]. During pretreatment, bright-field micrographs were acquired to document morphological changes using a TCM400 microscope (Labomed Inc., Los Angeles, CA, USA).

### 4.3. Morphometric Analysis of Lipid Droplets

To evaluate the effect of the inhibitor treatment on the adipogenic phenotype, intracellular lipid droplets were assessed as an indicator of adipocyte maturation. For this purpose, five adipocytes per experimental group were randomly selected from the day 14 micrographs. Lipid droplets within these cells were delineated as individual regions of interest for measurement of area and Feret’s diameter. The equivalent radius was calculated from the droplet area, and the number of lipid droplets per preadipocyte, along with their morphometric parameters, was measured using Fiji software (version 2.16, RRID: SCR_002285). To minimize bias, image files were anonymized, and the analysis was performed by a single operator who was blinded to the experimental conditions.

### 4.4. Immunocytochemistry of H3K9ac and H3K9me2

To evaluate the nuclear distribution of H3K9ac and H3K9me2, hPDLSCs were seeded in 24-well culture plates containing 18 mm diameter coverslips (1.2 × 10^4^ cells per well). After 24 h, the cells were treated with 100 nM TSA for 72 h. H3K9ac was assessed at the end of pretreatment, whereas both H3K9ac and H3K9me2 were analyzed on day 14 of adipogenesis, a time point selected based on previous findings in this model [19]. Cells were fixed with 4% paraformaldehyde (Sigma-Aldrich, St. Louis, MO, USA). For immunofluorescence, cells were blocked with 3% PBS-BSA and incubated overnight at 4° C with a rabbit anti-H3K9ac antibody (1:1000; ABclonal Technology, Wuhan, China; Cat# A21107, RRID: AB_3714809) or rabbit anti-H3K9me2 antibody (1:500; Millipore Sigma, Burlington, MA, USA; Cat# 07-441, RRID: AB_11212297). Subsequently, cells were incubated with donkey anti-rabbit IgG (H + L) Alexa Fluor 488 (Cat# A-21206; Invitrogen, Thermo Fisher Scientific, Waltham, MA, USA). Nuclei were counterstained with DAPI (1:1500; Sigma-Aldrich, St. Louis, MO, USA), and coverslips were mounted using ProLong Diamond Antifade Mountant (Thermo Fisher Scientific, Waltham, MA, USA).

Fluorescence intensity was analyzed using epifluorescence microscopy (Eclipse Ti2, Nikon Corporation, Tokyo, Japan) and confocal microscopy (Stellaris 5, Leica Microsystems, Wetzlar, Germany). For nuclear fluorescence intensity quantification, images were processed using Fiji software v2.16. The DAPI channel was used to segment nuclei using the StarDist 2D plugin [54] with TensorFlow [55] and CSBDeep [56] dependencies. The resulting regions of interest were applied to the corresponding fluorescence channel to measure fluorescence intensity per nuclear area. Data were analyzed using GraphPad Prism v10.3.1 (GraphPad Software, Boston, MA, USA).

### 4.5. Chromatin Immunoprecipitation

ChIP-seq analysis was performed on day 14 of adipogenic differentiation following the methodology established by Montero et al. [16]. Briefly, hPDLSCs cultured in T75 flasks (1.8 × 10^6^ cells per condition) were processed as one biological sample per experimental group (untreated and TSA) for initial genomic mapping. Each library was prepared and sequenced once per condition. Detailed quality metrics are provided in Figure S1.

Cells were fixed with 1% formaldehyde for 10 min and neutralized with 1.25 M glycine. Chromatin was fragmented using a Cole-Parmer CV18 sonicator (Cole-Parmer, Vernon Hills, IL, USA) for 30 cycles (60 s ON, 30 s OFF) to obtain fragments of approximately 100–500 bp, with fragment size verified by agarose gel electrophoresis. Chromatin was immunoprecipitated with 2.2 µg of rabbit anti-H3K9ac antibody (Invitrogen, Waltham, MA, USA; Cat# 49-1009; RRID: AB_2533860) in a final volume of 1 mL of dilution buffer and incubated overnight at 4 °C. The antibody used for ChIP is validated by the manufacturer for this application. Immune complexes were recovered with 50% protein G-agarose (Invitrogen, Waltham, MA, USA) and processed by washing, elution, reversal of cross-linking, and purification of the immunoprecipitated DNA. The DNA pellet was washed with 70% ethanol, air-dried, resuspended in 1X TE, and stored at −80 °C until further use.

### 4.6. Bioinformatics Analysis

ChIP-seq datasets have been deposited in the Gene Expression Omnibus (GEO) under accession number GSE346917.

#### 4.6.1. Library Preparation and Sequencing

Immunoprecipitated DNA was sent to Novogene Co., Ltd. (Beijing, China) for library construction and sequencing. Libraries were prepared using end repair, adapter ligation, and PCR amplification. Sequencing was performed on an Illumina NovaSeq 6000 platform (Illumina, San Diego, CA, USA) to generate 150 bp paired-end reads. Sequence data were obtained as compressed FASTQ files. One ChIP library per condition was constructed and sequenced. Raw sequencing depths before mapping and deduplication were 57,590,706 reads for the control condition and 47,876,980 reads for the TSA condition.

#### 4.6.2. Sequencing Read Processing

FASTQ files from the vehicle (control), TSA, and input conditions were aligned to the GRCh38/hg38 reference assembly using Bowtie2 v2.5.4 [57], using paired-end mapping and excluding mixed and discordant alignments. BAM files were sorted and indexed using Samtools v1.22.1 [58]. Read quality was evaluated using FastQC v0.12.1 [59] and mapping metrics were assessed with Samtools flagstat. Peak calling was performed using q = 0.001 as the significance threshold in MACS3 v3.0.3 [60]. Detailed quality parameters are presented in Figure S1.

#### 4.6.3. Global Library Quality Control

The quality and comparability of ChIP-seq libraries were assessed using sorted, indexed, and deduplicated BAM files, after filtering for mapping quality (MAPQ ≥ 10) and excluding GRCh38/hg38 blacklist regions. Global coverage and signal distribution were examined using deepTools v3.5.6 [61]. Global similarity among libraries was evaluated using principal component analysis (PCA). A bin-based count matrix was generated using multiBamSummary and visualized with plotPCA under the same filtering criteria. Detailed results are shown in Figure S2.

#### 4.6.4. Genomic Coverage

To visualize the genomic distribution of the H3K9ac signal, sorted BAM files were processed using deepTools v3.5.6 bamCoverage to generate counts-per-million (CPM)-normalized BigWig tracks, excluding GRCh38/hg38 blacklist regions. Signal at promoter regions was evaluated using TSS-centered matrices within a ±1.0 kb window, generated with computeMatrix and visualized as coverage profiles using plotProfile and as heatmaps using plotHeatmap. The distribution of H3K9ac peaks relative to genomic features was annotated using ChIPseeker v1.46.1 [62] and represented as stacked bar charts.

#### 4.6.5. Proximity-Based Gene Association

The proximity-based association of genes with H3K9ac-enriched regions was determined using GREAT v4.0.4 [63] and BED files derived from peak calling. Analysis was performed using the Basal plus extension model, with a basal regulatory domain of 5 kb upstream and 1 kb downstream of the TSS and a maximum extension of 500 kb in both directions for the association of distal regions with genes. Region-gene associations and distances to the TSS were recorded.

#### 4.6.6. Differential H3K9ac Enrichment

To evaluate differential enrichment between conditions, mapping depth was assessed using Samtools v1.22.1 flagstat. To normalize coverage, the control dataset was downsampled to 70% of its original depth. BAM files were analyzed using MACS3 v3.0.3 callpeak (-f BAMPE; -q = 0.001). The resulting bedGraph files from each condition were analyzed using MACS3 bdgdiff to identify differentially enriched regions, using a likelihood-ratio cutoff of -c 3.0, a minimum region length of -l 200 bp, and a maximum gap of -g 100 bp. Regions with higher enrichment in the vehicle or TSA condition, as well as shared regions, were identified.

#### 4.6.7. Regulatory Annotation of Differential Regions

Differential regions were classified according to their regulatory context by intersecting BED files with promoter and enhancer annotations in hg38 using bedtools v2.31.1 [64]. Promoters were defined using GENCODE v49 annotation with regions extending ±1.0 kb from the TSS. Enhancers were obtained from the ENCODE candidate cCRE registry via SCREEN, specifically including regions classified as enhancer-like signatures for the H9 cell line. For visual inspection of individual loci, additional ENCODE cCRE categories were included: PLS, proximal enhancer-like signatures (pELS), distal enhancer-like signatures (dELS), and CTCF-only binding sites. For each differential peak, this analysis identified its annotated regulatory context (promoter or enhancer) and, for promoters, the associated gene.

#### 4.6.8. Association Between Distal Regions and Promoters

To link distal regions to potential target genes, differentially enriched enhancers showing higher enrichment in the TSA condition were associated with the nearest differentially enriched promoter using the bedtools v2.31.1 closest function. Direct overlaps between intervals were excluded, and the genomic distance between each enhancer and its nearest promoter was recorded (-d -k 1 -io). Enhancer and promoter BED files were first sorted and filtered to remove redundancies. Subsequently, the identified promoters were intersected with the promoter annotation to append the corresponding gene annotation. This analysis established cis-linear associations between distal regions and promoters, together with their genomic distances (Figure S3).

#### 4.6.9. In Silico Functional Analysis

To identify overrepresented biological functions, genes annotated to differentially enriched promoters with higher enrichment in the TSA condition and genes linked to the promoter-enhancer (prom-enh) network under the same condition were analyzed as separate sets. Each set underwent functional enrichment analysis using ShinyGO v0.85 [65], with the false discovery rate (FDR) used as the significance threshold. Results were visualized as enrichment networks using STRING v12.0 [66].

#### 4.6.10. Selection of Candidate Genes

To prioritize candidate genes for downstream analysis, we integrated the gene sets described above: genes associated with differentially enriched promoters with higher enrichment in the TSA condition and genes linked to the prom-enh network under TSA. Selection was based on STRING interaction scores (score ≥ 0.400), Gene Ontology enrichment (FDR < 0.05), and statistical weight in the differential analysis (score > 3.0). Genes associated with biological processes related to adipogenesis, lipid metabolism, and epigenetic regulation were selected.

#### 4.6.11. Genomic Distribution Maps

To examine the local distribution of the H3K9ac signal across selected candidate genes, visualization was performed in the University of California, Santa Cruz (UCSC) Genome Browser and the hg38/GRCh38 assembly. CPM-normalized BigWig coverage tracks for the vehicle, TSA, and input conditions were loaded alongside the UCSC CpG island annotation. Quantitative evaluation of differential H3K9ac enrichment was performed by generating relative change coverage profiles (bigwigCompare, --operation log2 --pseudocount 0.1).

### 4.7. Statistical Analysis

Immunofluorescence and lipid droplet quantification experiments were performed with three independent biological replicates, with technical duplicates within each experiment. ChIP-seq was performed with one biological sample per condition; therefore, biological variability could not be estimated for this assay, and the results are presented as initial genomic mapping. Results are expressed as the mean ± standard deviation, and differences between conditions were analyzed using an unpaired two-tailed Student’s *t*-test in GraphPad Prism v.10.3.1. Differences were considered significant at *p* < 0.05. For ChIP-seq, enriched regions were identified using MACS3 v3.0.3 callpeak. To evaluate genomic enrichment, the local background signal was modeled using a dynamic Poisson with reference to the input hPDLSCs. Significantly enriched regions were identified using a threshold of q = 0.001.

## 5. Conclusions

TSA pretreatment is associated with changes in the adipogenic phenotype and alterations in H3K9ac and H3K9me2, facilitating H3K9ac enrichment at promoters linked to the adipogenic program in hPDLSCs. Together, these findings support H3K9ac as a relevant epigenetic feature associated with adipogenic priming and provide an initial genomic framework for identifying regulatory regions and candidate genes that may inform subsequent functional validation and targeted epigenetic studies in regenerative and metabolic research.

## Figures and Tables

**Figure 1:**
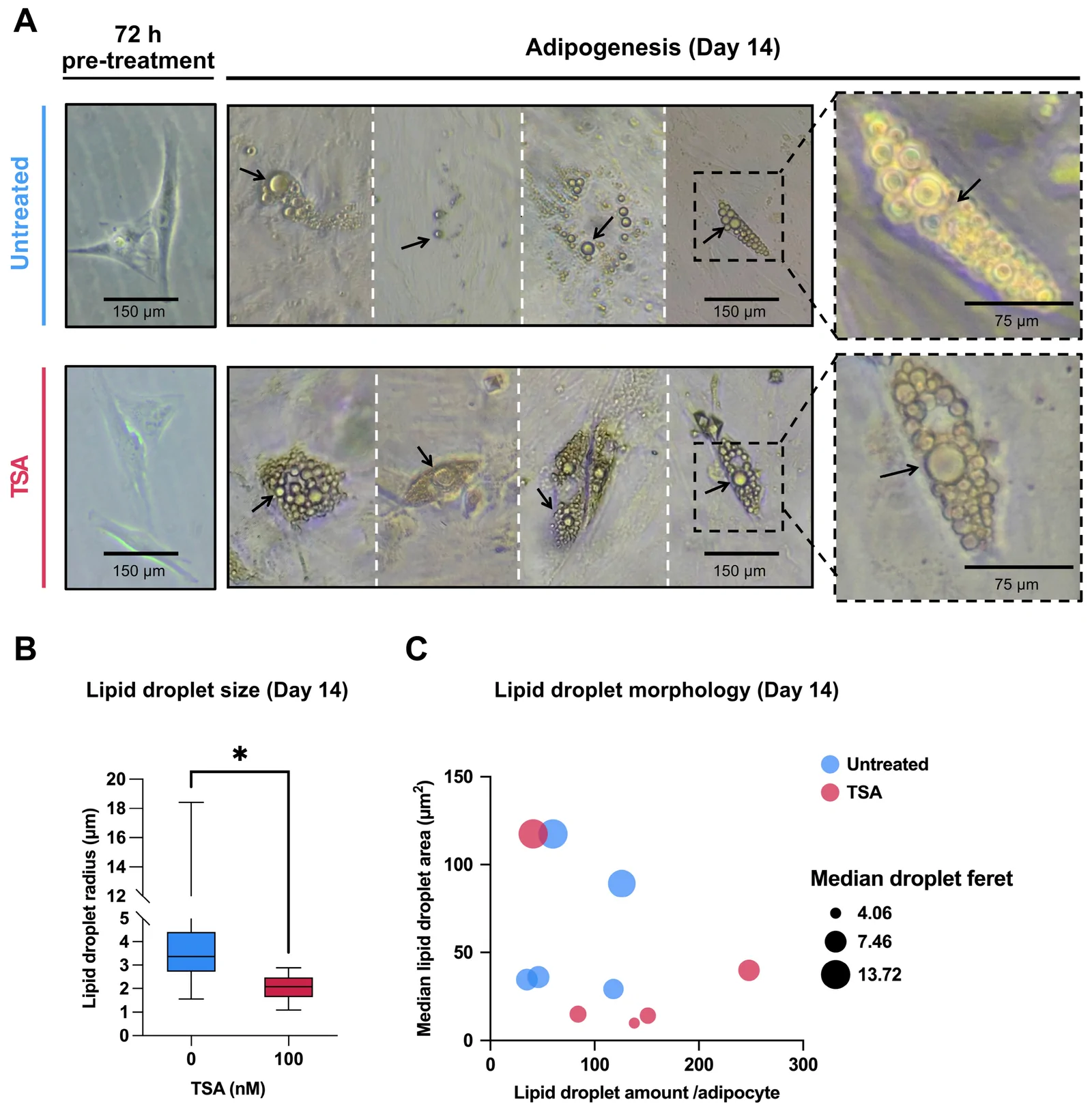
Cell morphology and lipid droplet analysis during adipogenesis in the absence or presence of trichostatin A (TSA) in human periodontal ligament stem cells (hPDLSCs) at day 14. (**A**) Bright-field micrographs of hPDLSCs undergoing adipogenic differentiation under untreated conditions following pretreatment with TSA; images obtained 72 h after treatment are shown in the bottom row. The upper panels correspond to magnified views of the regions outlined by dashed boxes, and arrows indicate representative lipid droplets. Scale bars: 150 µm and 75 µm. (**B**) Box plot showing the distribution of the equivalent radii of individual lipid droplets, presented to illustrate biological variance. To prevent pseudoreplication, statistical significance (* *p* < 0.05) was determined using an unpaired two-tailed Student’s *t*-test based on the arithmetic means of three independent biological replicates (n = 3). (**C**) Multivariable representation of the adipogenic phenotype. The x-axis indicates the number of lipid droplets per cell, the y-axis indicates the median lipid droplet area, and bubble size represents the median Feret’s diameter. Colors indicate the experimental conditions.

**Figure 2:**
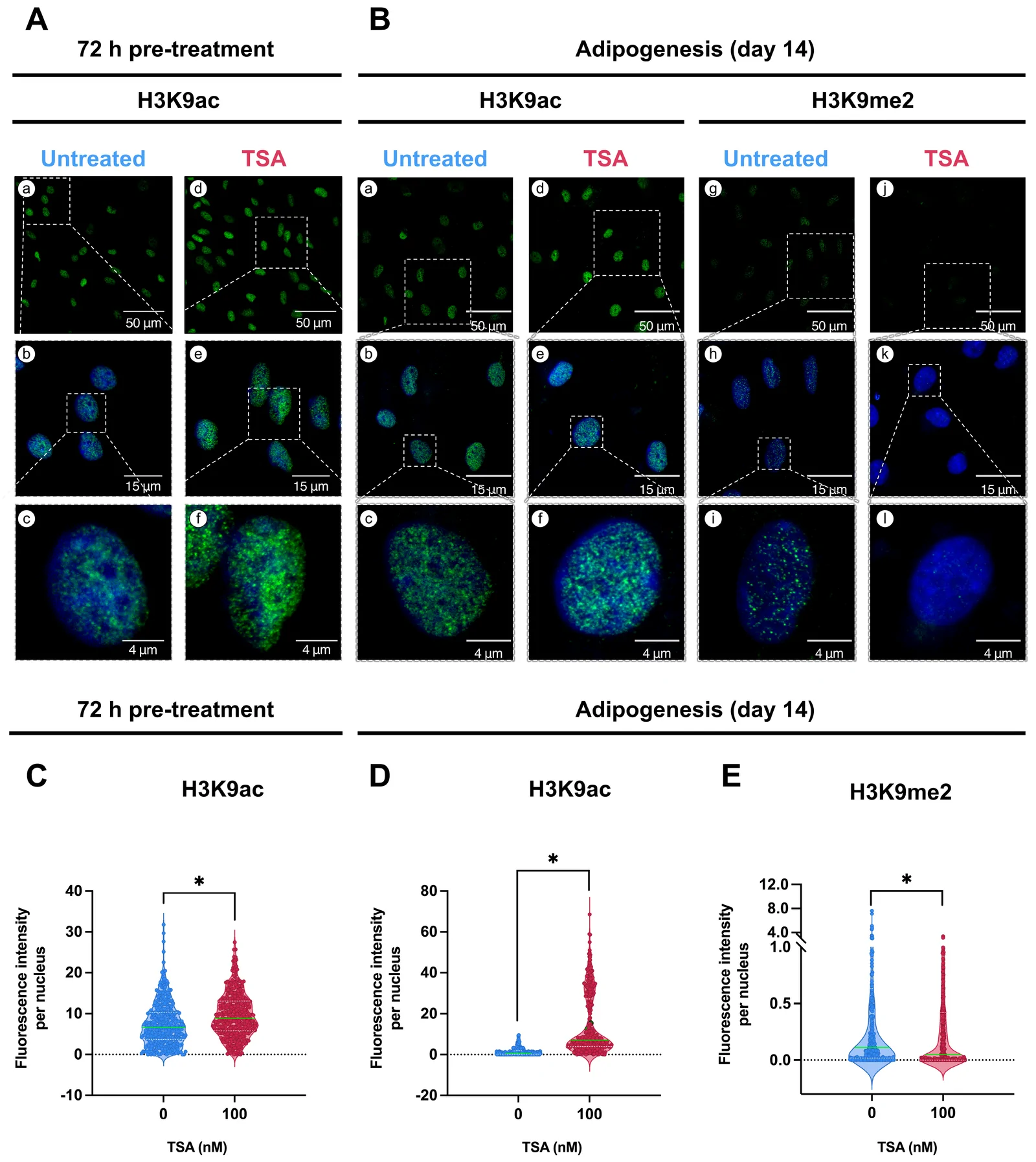
Analysis of the nuclear distribution of histone 3 lysine 9 acetylation (H3K9ac) and histone H3 lysine 9 dimethylation (H3K9me2) during hPDLSC adipogenesis following TSA exposure. (**A**) Confocal microscopy images of H3K9ac (Alexa Fluor 488, green) and 4′,6-diamidino-2-phenylindole (DAPI) nuclear counterstaining (blue) without treatment and after a 72 h exposure to 100 nM TSA. (**B**) Confocal microscopy images of H3K9ac and H3K9me2 (Alexa Fluor 488, green) with DAPI nuclear counterstaining (blue) at day 14 of adipogenesis without prior exposure and after 72 h of 100 nM TSA treatment. (**C**) Violin plot showing the distribution of nuclear fluorescence intensities for H3K9ac after 72 h in the untreated and TSA-treated groups. (**D**,**E**) Violin plots showing the distributions of nuclear fluorescence intensity of H3K9ac and H3K9me2 in both experimental groups at day 14. To visualize the distribution of fluorescence intensities, 378 nuclei per condition, derived from three independent experiments (126 nuclei per replicate), were plotted. Statistical analyses were performed using the three independent biological replicates as experimental units. Comparisons between conditions were performed using an unpaired two-tailed Student’s *t*-test with a significance threshold of * *p* < 0.05. Scale bars: 50 µm, 15 µm, and 4 µm.

**Figure 3:**
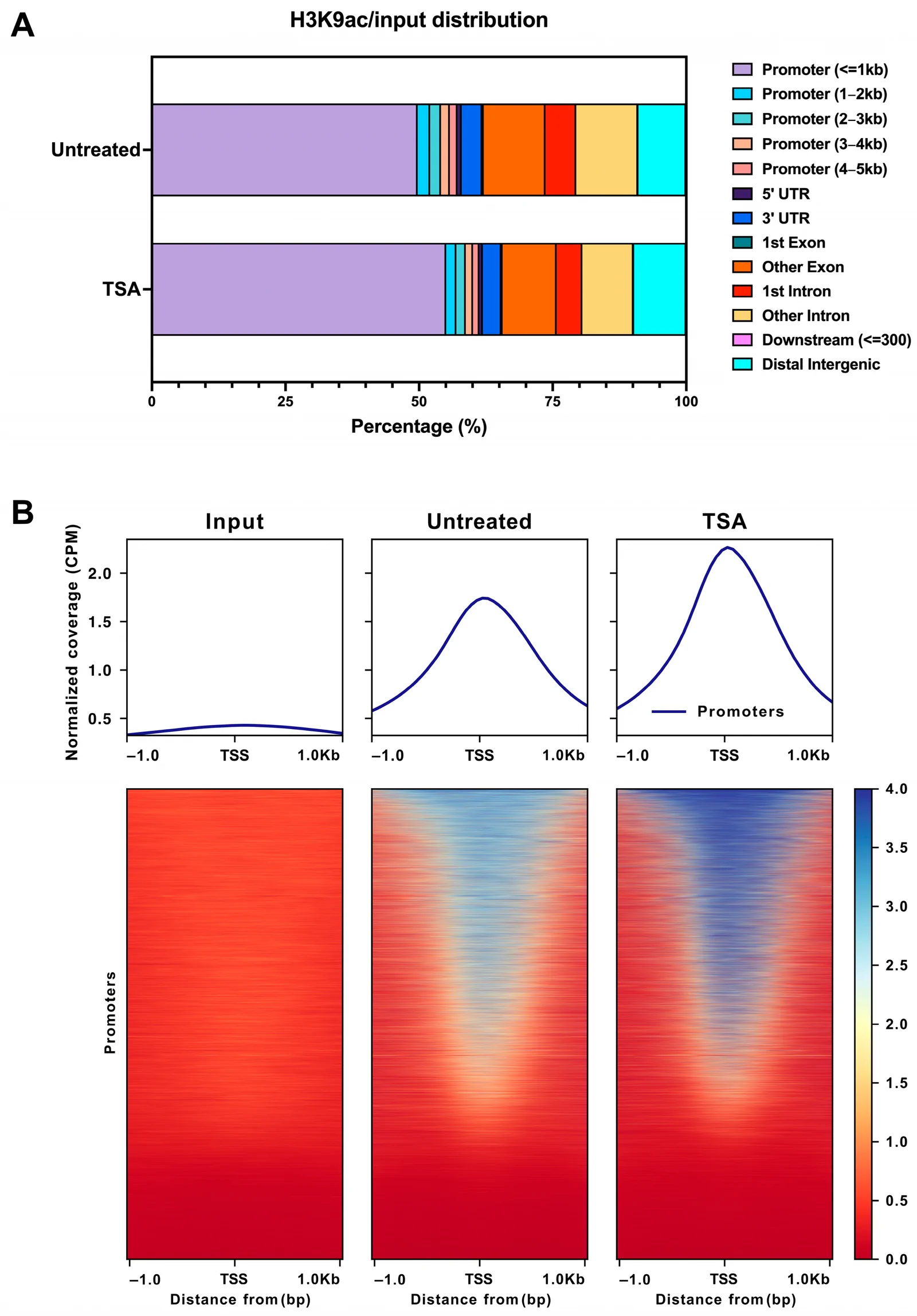
Global genomic distribution of H3K9ac and enrichment at promoter regions. (**A**) Stacked bar plot showing the proportion of peaks annotated to different genomic categories in the untreated and TSA groups. (**B**) Average normalized coverage profiles and heatmaps of the H3K9ac signal centered on transcription start sites (TSS) in the input, untreated, and TSA samples within a ±1.0 kb window. The color scale indicates normalized coverage intensity.

**Figure 4:**
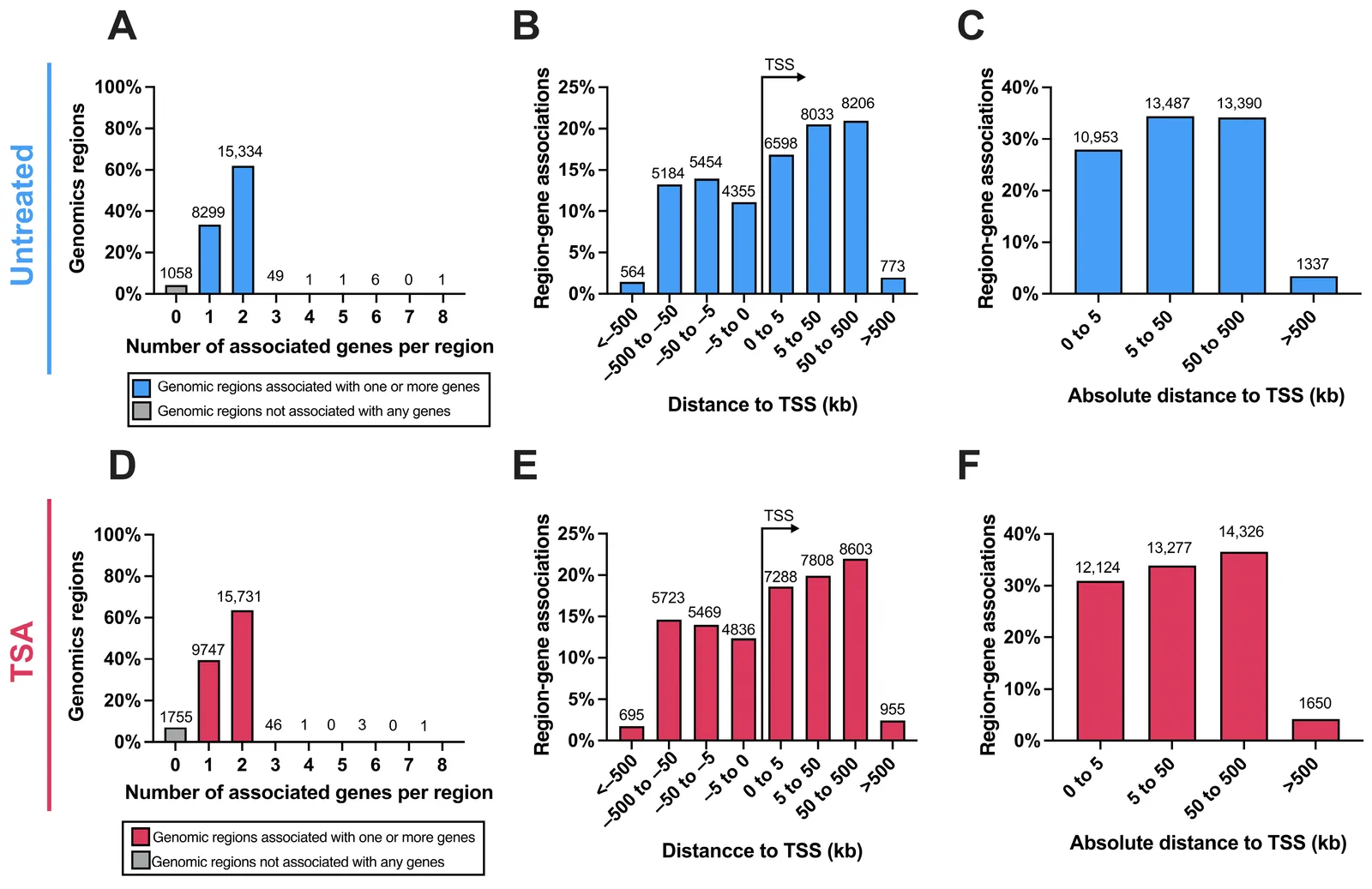
Genomic proximity-based association analysis of differential H3K9ac regions using GREAT. Gene association and distance-based analyses of H3K9ac peaks from hPDLSCs at day 14 of adipogenesis are shown for the (**A**–**C**) untreated group (blue) and the (**D**–**F**) TSA group (red), normalized to input. For each condition, counts for the represented categories and the total number of associated regions included in the analysis are shown. Gene association was performed using GREAT v4.0.4 and the Basal plus extension model.

**Figure 5:**
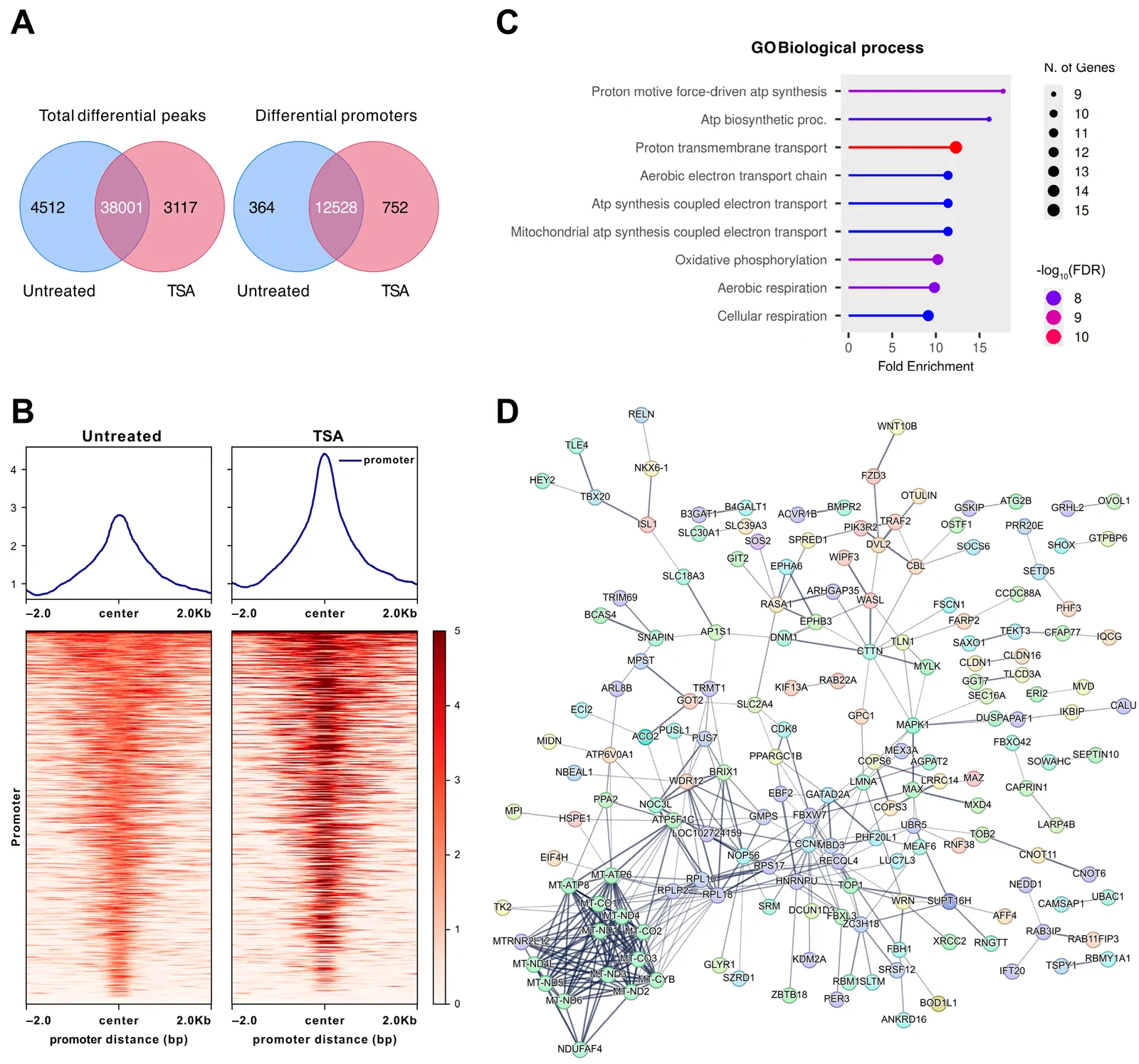
Differential H3K9ac enrichment analysis between the untreated and TSA conditions. (**A**) Venn diagrams illustrating the distribution of differential regions obtained using MACS3 bdgdiff, including the total number of differential peaks and the subset corresponding to differentially enriched promoters in the untreated and TSA conditions. (**B**) Average profiles and heatmaps of the H3K9ac signal across differentially enriched promoters, centered on the midpoint of each region within a ±2.0 kb window in both the untreated and TSA conditions. The color scale indicates signal intensity. (**C**) Functional enrichment plot of selected terms associated with genes linked to differentially enriched promoters. The x-axis represents fold enrichment, dot size indicates the number of genes, and color represents –(Log_10_) (FDR). (**D**) Schematic representation of a promoter relative to the TSS and the protein–protein interaction network generated using STRING from genes associated with differentially enriched promoters in the TSA condition.

**Figure 6:**
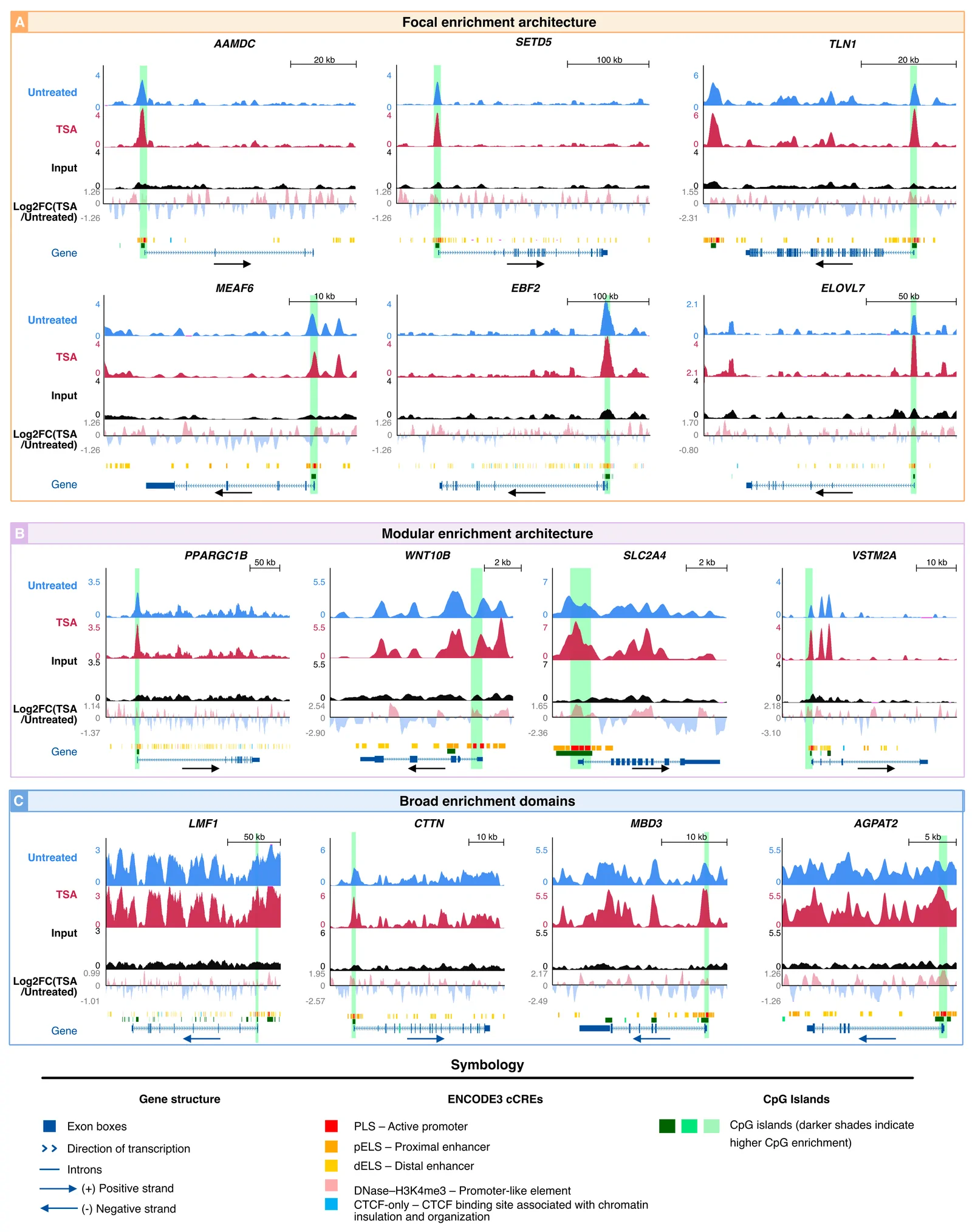
Representative local architectures of H3K9ac enrichment at loci associated with adipogenesis, energy metabolism, and chromatin remodeling. Representative genome browser views showing local H3K9ac enrichment patterns at selected loci in hPDLSCs. Based on visual inspection of the enrichment profiles, three descriptive architectures were recognized: (**A**) dominant focal peak architecture, characterized by a single predominant enrichment region restricted to the 5′ end; (**B**) segmented enrichment architecture, characterized by multiple discrete and consecutive enrichment regions distributed across the locus; and (**C**) broad enrichment domains, characterized by extended regions of H3K9ac enrichment spanning large portions of the locus. Representative genes shown include *AAMDC*, *SETD5*, *TLN1*, *MEAF6*, *EBF2*, *ELOVL7*, *PPARGC1B*, *WNT10B*, *SLC2A4*, *VSTM2A*, *LMF1*, *CTTN*, *MBD3*, and *AGPAT2*. Genome browser tracks include H3K9ac chromatin immunoprecipitation sequencing (ChIP-seq) signals (untreated and TSA), the quantitative differential enrichment track (Log2FC(TSA/Untreated)), Reference Sequence (RefSeq) gene annotation, CpG islands, and candidate cis-regulatory elements (cCREs) annotated by the ENCODE Registry, including PLS, pELS, dELS, DNase–H3K4me3, and CTCF-only: CTCF binding sites associated with chromatin insulation and organization. Images were generated using the UCSC Genome Browser.

## Data Availability

The data presented in this study are available from the corresponding author. The ChIP-seq datasets generated during the current study are openly available in the Gene Expression Omnibus (GEO) repository under the accession number GSE346917 publicly accessible at https://www.ncbi.nlm.nih.gov/geo/query/acc.cgi?acc=GSE346917 (accessed on 1 September 2026). Additionally, a review token is provided for confidential data access prior to public release: enter token mtuzuiqgdnibfiz into the box.

## Supplementary Materials

The following supporting information can be downloaded at https://www.mdpi.com/article/10.3390/ijms27219898/s1.

## Abbreviations

The following abbreviations are used in this manuscript:

hPDLSCs	Human Periodontal Ligament Stem Cells
ChIP-seq	Chromatin Immunoprecipitation Sequencing
H3K9ac	Histone H3 lysine 9 acetylation
H3K9me2	Histone H3 lysine 9 dimethylation
HDACs	Histone Deacetylases
MAPQ	Mapping Quality
CPM	Counts Per Million
TSS	Transcription Start Site
PCA	Principal Component Analysis
FDR	False Discovery Rate
cCREs	Candidate Cis-Regulatory Elements
ELS	Enhancer-like Signatures
PLS	Promoter-like Signatures
pELS	Proximal Enhancer-like Signatures
dELS	Distal Enhancer-like Signatures
CTCF	CCCTC-binding Factor
IQR	Interquartile Range
SLC2A4	Solute Carrier Family 2 Member 4
AAMDC	Adipogenesis Associated MKL1 Cofactor
SETD5	SET Domain Containing 5
ELOVL7	ELOVL Fatty Acid Elongase 7
MEAF6	MYST/Esa1-Associated Factor 6
EBF2	EBF Transcription Factor 2
TLN1	Talin 1
PPARGC1B	PPARG Coactivator 1 Beta
WNT10B	Wnt Family Member 10B
VSTM2A	V-set and Transmembrane Domain Containing 2A
LMF1	Lipase Maturation Factor 1
CTTN	Cortactin
MBD3	Methyl-CpG Binding Domain Protein 3
AGPAT2	1-Acylglycerol-3-Phosphate O-Acyltransferase 2
MAPK1	Mitogen-Activated Protein Kinase 1
