# Supplementary material for: Trichostatin A-Induced H3K9ac Hyperacetylation Selectively Enriches Promoters in Adipogenic Human Periodontal Ligament Stem Cells
Source: Int J Mol Sci. 2026 Nov 5;27(21):9898. doi: 10.3390/ijms27219898 (PMC13764430; doi:10.3390/ijms27219898)
Supplement: Supplementary file 1 [file ijms-27-09898-s001.zip › Table S1.pdf]

"Trichostatin A-induced H3K9ac hyperacetylation reshapes the genomic landscape following adipogenic induction in human periodontal ligament stem cells"

(Manuscript ID: ijms-4596243-Major Revisions)

Table S1: Gene Ontology enrichment results from ShinyGO, detailing the specific genes mapped to each pathway for differential promoters under TSA conditions.

Enrichment FDR	nGenes	Pathway Genes	Fold Enrichment	Pathways (click for details)	Genes
**5.6E-09**	9	94	17.7	Proton motive force-driven atp synthesis	MT-ATP6 MT-ATP8 MT-ND6 MT-ND2 MT-ND5 MT-ND3 MT-ND4 MT-ND1 MT-ND4L
**1.6E-08**	9	122	16.1	Atp biosynthetic proc.	MT-ATP6 MT-ATP8 MT-ND6 MT-ND2 MT-ND5 MT-ND3 MT-ND4 MT-ND1 MT-ND4L
**2.4E-08**	9	148	14.8	Nucleoside triphosphate biosynthetic proc.	MT-ATP6 MT-ATP8 MT-ND6 MT-ND2 MT-ND5 MT-ND3 MT-ND4 MT-ND1 MT-ND4L
**2.4E-08**	9	134	14.8	Purine nucleoside triphosphate biosynthetic proc.	MT-ATP6 MT-ATP8 MT-ND6 MT-ND2 MT-ND5 MT-ND3 MT-ND4 MT-ND1 MT-ND4L
**2.4E-08**	9	139	14.8	Ribonucleoside triphosphate biosynthetic proc.	MT-ATP6 MT-ATP8 MT-ND6 MT-ND2 MT-ND5 MT-ND3 MT-ND4 MT-ND1 MT-ND4L
**2.4E-08**	9	133	14.8	Purine ribonucleoside triphosphate biosynthetic proc.	MT-ATP6 MT-ATP8 MT-ND6 MT-ND2 MT-ND5 MT-ND3 MT-ND4 MT-ND1 MT-ND4L
**2.4E-08**	9	274	14.8	Atp metabolic proc.	MT-ATP6 MT-ATP8 MT-ND6 MT-ND2 MT-ND5 MT-ND3 MT-ND4 MT-ND1 MT-ND4L
**1.7E-07**	9	297	12.6	Purine ribonucleoside triphosphate metabolic proc.	MT-ATP6 MT-ATP8 MT-ND6 MT-ND2 MT-ND5 MT-ND3 MT-ND4 MT-ND1 MT-ND4L
**1.1E-11**	15	221	12.3	Proton transmembrane transport	OTOP1 MT-ND5 MT-ND4 MT-ATP6 SLC18A3 MT-ND6 MT-CO2 MT-CYB MT-ND2 MT-CO1 MT-ND3 MT-ND1 MT-CO3 MT-ND4L MT-ATP8
**3.6E-07**	9	320	11.8	Nucleoside triphosphate metabolic proc.	MT-ATP6 MT-ATP8 MT-ND6 MT-ND2 MT-ND5 MT-ND3 MT-ND4 MT-ND1 MT-ND4L
**3.6E-07**	9	304	11.8	Purine nucleoside triphosphate metabolic proc.	MT-ATP6 MT-ATP8 MT-ND6 MT-ND2 MT-ND5 MT-ND3 MT-ND4 MT-ND1 MT-ND4L
**2.0E-08**	11	110	11.4	Aerobic electron transport chain	MT-CYB MT-ND2 MT-CO3 MT-ND6 MT-ND5 MT-ND3 MT-ND4 MT-ND1 MT-CO2 MT-CO1 MT-ND4L
**2.0E-08**	11	117	11.4	Atp synthesis coupled electron transport	MT-CO2 MT-CYB MT-ND2 MT-CO3 MT-ND6 MT-ND5 MT-ND4 MT-ND4L MT-ND3 MT-ND1 MT-CO1
**2.0E-08**	11	117	11.4	Mitochondrial atp synthesis coupled electron transport	MT-CYB MT-ND2 MT-CO3 MT-ND6 MT-ND5 MT-ND3 MT-ND4 MT-ND1 MT-CO2 MT-CO1 MT-ND4L

**2.7E-08**	11	132	10.8	Respiratory electron transport chain	MT-CO2 MT-CYB MT-ND2 MT-CO1 MT-CO3 MT-ND6 MT-ND5 MT-ND4 MT-ND4L MT-ND3 MT-ND1
**5.6E-09**	13	165	10.2	Oxidative phosphorylation	MT-CO2 MT-CYB MT-ND2 MT-CO3 MT-ATP6 MT-ATP8 MT-ND6 MT-ND5 MT-CO1 MT-ND4 MT-ND4L MT-ND3 MT-ND1
**8.6E-09**	13	323	9.8	Aerobic respiration	MT-CO2 MT-CYB MT-ND2 MT-CO1 MT-ND4 MT-ND1 MT-CO3 MT-ATP6 MT-ATP8 MT-ND6 MT-ND5 MT-ND4L MT-ND3
**9.7E-08**	11	144	9.8	Electron transport chain	MT-CO2 MT-CYB MT-ND2 MT-CO1 MT-CO3 MT-ND6 MT-ND5 MT-ND4 MT-ND4L MT-ND3 MT-ND1
**2.0E-08**	13	362	9.1	Cellular respiration	MT-CO2 MT-CYB MT-ND2 MT-CO1 MT-ND4 MT-ND1 MT-CO3 MT-ATP6 MT-ATP8 MT-ND6 MT-ND5 MT-ND4L MT-ND3
