# Supplementary material for: Trichostatin A-Induced H3K9ac Hyperacetylation Selectively Enriches Promoters in Adipogenic Human Periodontal Ligament Stem Cells
Source: Int J Mol Sci. 2026 Nov 5;27(21):9898. doi: 10.3390/ijms27219898 (PMC13764430; doi:10.3390/ijms27219898)
Supplement: Supplementary file 1 [file ijms-27-09898-s001.zip › Table S2.pdf]

"Trichostatin A-induced H3K9ac hyperacetylation reshapes the genomic landscape following adipogenic induction in human periodontal ligament stem cells"

(Manuscript ID: ijms-4596243-Major Revisions)

Table S2: H3K9ac enrichment (Log2FC) at candidate gene promoters upon TSA treatment.

**Promoter -Gene Locus**	**Log2FC (TSA vs Untreated)**
AAMDC	0.88971
SETD5	0.78993
TLN1	1.07491
MEAF6	0.87160
EBF2	0.52325
ELOVL7	1.11471
PPARGC1B	0.80610
WNT10B	1.55009
SLC2A4	1.60045
VSTM2A	1.82998
LMF1	0.46687
CTTN	1.03728
MBD3	1.12870
AGPAT2	1.26776
