# Supplementary material for: Trichostatin A-Induced H3K9ac Hyperacetylation Selectively Enriches Promoters in Adipogenic Human Periodontal Ligament Stem Cells
Source: Int J Mol Sci. 2026 Nov 5;27(21):9898. doi: 10.3390/ijms27219898 (PMC13764430; doi:10.3390/ijms27219898)
Supplement: Supplementary file 1 [file ijms-27-09898-s001.zip › Table S3.pdf]

“Trichostatin A-induced H3K9ac hyperacetylation reshapes the genomic landscape following adipogenic induction in human periodontal ligament stem cells”

(Manuscript ID: ijms-4596243-Major Revisions)

Table S3: Absolute counts and genomic distribution of identified peaks across distinct genomic features for Untreated, TSA-specific, and Common datasets.

Differential peaks annotations			
	Untreated	TSA	Common
**Promoter <=1kb**	364	752	12528
**Exon**	1461	1510	31492
**intron**	3820	1809	36271
**intergenic**	778	906	4173
**Five prime UTR**	257	608	12977
**Three prime UTR**	341	111	3912

Absolute counts of H3K9ac-enriched regions per genomic feature category. "Untreated" and "TSA" denote regions with higher enrichment in each condition; "Common" denotes regions enriched in both. A Chi-square test confirmed a nonrandom distribution (χ^2 = 996.1, df = 5, p < 0.000001). Abbreviations: H3K9ac, histone H3 lysine 9 acetylation; TSA, trichostatin A; UTR, untranslated region.
